# High sensitivity mapping of brain-wide functional networks in awake mice using simultaneous multi-slice fUS imaging

**DOI:** 10.1162/imag_a_00030

**Published:** 2023-11-15

**Authors:** Adrien Bertolo, Jeremy Ferrier, Silvia Cazzanelli, Samuel Diebolt, Mickael Tanter, Sophie Pezet, Mathieu Pernot, Bruno-Félix Osmanski, Thomas Deffieux

**Affiliations:** Physics for Medicine Paris, ESPCI Paris, INSERM, CNRS, PSL Research University, Paris, France.; Iconeus, Paris, France.; Institute of Psychiatry and Neuroscience of Paris, INSERM, University of Paris, France

**Keywords:** volumetric imaging, functional ultrasound, brain imaging, visual pathway, functional connectivity, awake mice, connectomics

## Abstract

Functional ultrasound (fUS) has received growing attention in preclinical research in the past decade, providing a new tool to measure functional connectivity (FC) and brain task-evoked responses with single-trial detection capability in both anesthetized and awake conditions. Most fUS studies rely on 2D linear arrays to acquire one slice of the brain. Volumetric fUS using 2D matrix or row-column arrays has recently been demonstrated in rats and mice but requires invasive craniotomy to expose the brain due to a lack of sensitivity. In a previous study, we proposed the use of motorized linear arrays, allowing imaging through the skull in mice for multiple slices with high sensitivity. However, the tradeoff between the field of view and temporal resolution introduced by motorized scanning prevents acquiring brain-wide resting-state FC data with a sufficient volume rate for resting-state FC analysis. Here, we propose a new hybrid solution optimized and dedicated to brain-wide transcranial FC studies in mice, based on a newly developed multi-array transducer allowing simultaneous multi-slicing of the entire mouse cerebrum. We first demonstrate that our approach provides a better imaging quality compared to other existing methods. Then, we show the ability to image the whole mouse brain non-invasively through the intact skin and skull during visual stimulation under light anesthesia to validate this new approach. Significant activation was detected along the whole visual pathway, at both single and group levels, with more than 10% of augmentation of the cerebral blood volume (CBV) signal during the visual stimulation compared to baseline. Finally, we assessed resting-state FC in awake head-fixed animals. Several robust and long-ranged FC patterns were identified in both cortical and sub-cortical brain areas, corresponding to functional networks already described in previous fMRI studies. Together, these results show that the multi-array probe is a valuable approach to measure brain-wide hemodynamic activity in mice with an intact skull. Most importantly, its ability to identify robust resting-state networks is paving the way towards a better understanding of the mouse brain functional organization and its breakdown in genetic models of neuropsychiatric diseases.

## 1 Introduction

Functional ultrasound (fUS) imaging is a recent modality that is able to probe the brain activity at a high spatiotemporal resolution (Mace et al., 2013; B.-F. Osmanski et al., 2014). In a similar way to blood oxygen level-dependent functional magnetic resonance imaging (BOLD-fMRI), fUS imaging relies on the neurovascular coupling to infer brain activity indirectly from measurement of hemodynamic signals. This is achieved by combining multiple echoes from thousands of ultrasound plane waves emitted at an ultrafast frame rate. The resulting Power Doppler (PD) signal is directly proportional to the CBV, that is*,* the amount of blood flowing through the voxel at a point in time.

fUS is particularly suited for studying neural networks in the mouse brain, the most common preclinical model in neuroscience. Using an emission frequency of 15 MHz, fUS images yield an in-plane spatial resolution of 100 × 100 µm (and ~500 µm slice thickness). The direct visualization of blood flow through the Doppler effect also gives fUS imaging a remarkable sensitivity to hemodynamic variations and specifically to those of neuronal origin. A growing body of evidence is now showing that fUS signals faithfully report multi-unit neuronal activity in mice through the neurovascular coupling (Aydin et al., 2020; Boido et al., 2019; Nunez-Elizalde et al., 2022), despite open questions regarding single unit activity uncoupling in primates at rest (Claron et al., 2023).

Most importantly, fUS is seamlessly compatible with awake behaving animals, whether head-fixed (Ferrier et al., 2020; Macé et al., 2018) or freely moving (Rabut et al., 2020; Sieu et al., 2015; Tiran et al., 2017) thanks to the miniaturization of ultrasonic linear arrays (2D imaging) which can now be tethered to the animal’s head using an implanted frame. Hence, fUS has been successfully applied to characterize many task-based (Gesnik et al., 2017; Macé et al., 2011, 2018; B. F. Osmanski et al., 2014) and resting-state functional systems (Ferrier et al., 2020; B.-F. Osmanski et al., 2014; Rabut et al., 2020).

However, a main restriction compared to fMRI is the 2D limitation, due to the geometry of current ultrasonic probes (1D linear arrays). While sequential scanning of the entire brain has been proposed using a motorized stage, it involves long acquisition time (>2 h per animal) and repeated stimulus presentation at each position to cover the whole brain (Gesnik et al., 2017; Macé et al., 2018). As multiple sessions must be repeated at each position to capture the whole brain, one limitation of this method is also that the animal’s behavior may vary from one session to another, and thus across slices. Most importantly, this approach is incompatible with the study of resting-state functional connectivity networks as it is inherently based on the synchronous measure of spontaneous oscillatory activity within spatially distributed brain regions (Coletta et al., 2020). We recently introduced an alternative approach using fast plane-switching transducers to significantly reduce the scanning time (Bertolo, Nouhoum, et al., 2021). However, the tradeoff between the number of positions imaged and the time resolution limits the field of view to a section of a few millimeters only.

On the other hand, full-3D volumetric fUS has been successfully achieved in rodents using fully populated matrix (FPM) arrays and row-column addressing (RCA) transducers. However, this approach suffers from a significant lack of sensitivity, calling for a surgery to expose the brain (Brunner et al., 2020; Rabut et al., 2019; Sauvage et al., 2019). In this context, further developments are needed to improve the spatial coverage of fUS imaging to achieve a more global view of the brain without compromising on resolution, frame rate, or sensitivity.

Here, we introduce a new multi-array probe consisting of four combined linear arrays, allowing simultaneous multi-slice (SMS) fUS imaging of the mouse brain. This new type of transducer increases fUS time resolution by a factor of four without compromising on sensitivity and spatial resolution. We first show that the multi-array probe can achieve almost brain-wide coverage in mice at 100 × 100 × 525 µm in under 2.4 s, non-invasively through the skin and bone. By comparing the imaging quality across different methods on the same animal before and after craniotomy, we provide evidence that the multi-array probe is more sensitive than FPM and RCA transducers. We then validate the ability of SMS-fUS imaging to spatially map brain activity using a simple visual activation paradigm in lightly anesthetized animals, showing significant CBV increases in areas of the visual pathway. Finally, this approach is extended to resting-state functional connectivity mapping in awake head-fixed mice. Seed-based and multivariate analyses reveal reliable detection of bilateral connectivity, including long-range connections in both cortical and sub-cortical brain regions.

## 2 Materials and Methods

### 2.1 Ethics

Twelve male C57BL/6 mice (7-8 weeks old, Janvier Labs, France) were used with approval from our local ethics committee (Comité d’éthique en matière d’expérimentation animale number 59, “Paris Centre et Sud”, project #2017-23). Animals were housed four per cage with a 12 h light/dark cycle, a constant temperature at 22°C, and unlimited access to food and water. Before beginning the experiments, animals are given a 1-week minimum acclimatization period to housing conditions. All experiments have been performed in agreement with the European Community Council Directive of 22 September 2010 (010/63/UE).

Methods were carried out following relevant guidelines and regulations and in compliance with ARRIVE guidelines (Percie du Sert et al., 2020).

### 2.2 Visual stimulation experiments in lightly anesthetized mice

**Fig. 1.**
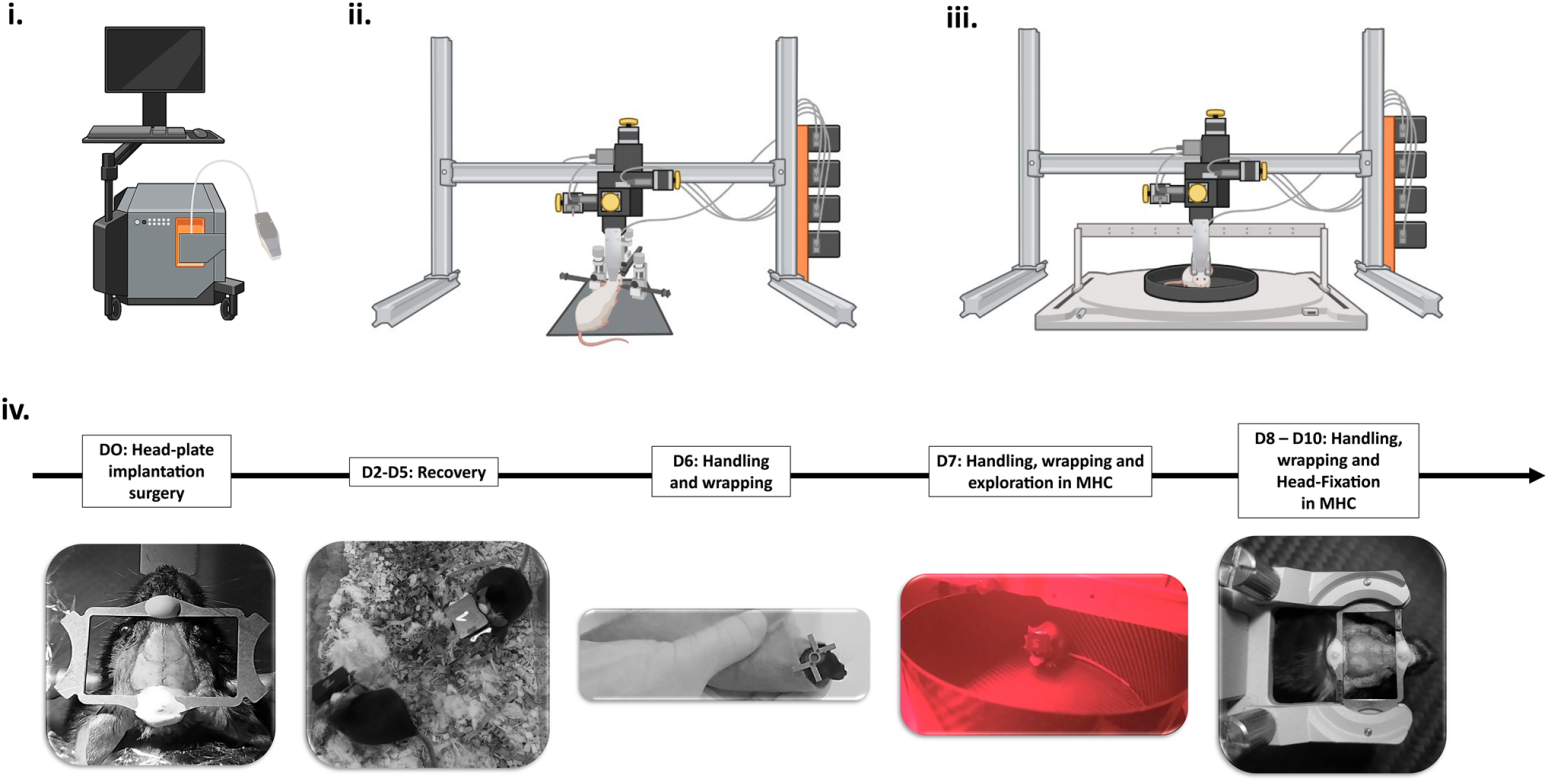
Experimental designs used for both anesthetized experiments (ii) and awake (iii, iv) experiments. (i) Iconeus One scanner (256 channels ultrasound system) driving the multi-array probe. (ii) Experimental setup for visual stimulation in lightly anesthetized mice. The anesthetized mouse is shaved and placed in a stereotaxic frame. The multi-array probe is mounted on a 4-axis motorized stage. A white LED is placed 30 cm from the mouse’s eyes for visual stimulation. (iii) Experimental setup for resting-state functional connectivity in awake mice. The multi-array probe is mounted on the 4-axis motor stage, and placed just above the head of the mouse, in the MHC. (iv) Different steps of the animal habituation protocol (post-surgery recovery, handling, wrapping for installation in the MHC, head-fixed exploration in the MHC). The first imaging session can be set at D11.

Visual stimulation experiments were performed in a dark room, with mice in a lightly anesthetized condition (Fig. 1(ii)). All acquisitions (one per animal, n = 6) were considered in the statistical analysis.

#### 2.2.1 Animal preparation

Mice were anesthetized by an initial intraperitoneal (IP) injection of ketamine and xylazine mixture at 100 mg/kg and 10 mg/kg, respectively. The hair was shaved using depilatory cream, and the mouse was positioned on a stereotaxic frame. Anesthesia was maintained with a low isoflurane supply throughout the imaging session (0.5% administered through a nose cone in an 80/20 air/oxygen stream). The eyes of the mouse were protected using an ointment (Ocry-gel, TVM, UK). Body temperature was controlled with a rectal probe connected to a heating pad set at 37°C. Respiration and heart rate were monitored using a PowerLab data acquisition system with the LabChart software (ADInstruments, USA). Additional IP ketamine/xylazine doses (25 mg/kg and 2.5 mg/kg, respectively) were infused intermittently (every 90 to 120 min), as deemed necessary based on changes in physiological parameters.

#### 2.2.2 Visual stimulation protocol

Visual stimulation was delivered using a white LED light positioned 30 cm from the mouse (measured luminance of 15 lux). After a baseline of 60 s, eight stimuli (30 s of flickered light consisting of 200 ms pulses with a 4 Hz frequency) were repeated every 90 s, resulting in a total acquisition duration of 780 s.

### 2.3 Resting-state functional connectivity in awake mice

Resting-state FC acquisitions (1200 s) were performed in awake head-fixed conditions (Fig. 1(iii)). Two animals (out of six) underwent a second imaging session as the rate of calm periods was considered as too short (more details can be found in Section 2.6).

#### 2.3.1 Surgical implantation of metal plate

A headpost with an imaging window of 13 × 21 mm² was surgically implanted for head fixation. The procedure has been described in detail previously (Bertolo, Nouhoum, et al., 2021). Briefly, mice were anesthetized using ketamine (100 mg/kg) and medetomidine (1 mg/kg). After hair shaving and skin disinfection, lidocaine was administered under the scalp, and the skin was excised. The head plate (Neurotar, Model 14) was attached to the skull using two anchoring screws and dental cement (Super-bond, C&B). The imaging window was sealed using Kwik-cast, and anesthesia was reversed by a subcutaneous atipamezole injection (1 mg/ml). Meloxicam (5 mg/kg, IP) was administered for postoperative pain, and the mice were recovered in their home cage.

#### 2.3.2 Habituation and training

A head-restrained imaging setup was used for awake imaging (Mobile HomeCage, Neurotar, Finland). The mice were head-fixed with a rigid metal clamp and positioned in a floating round carbon-fiber cage, allowing them to explore the environment freely. Six days after the surgery, mice were repeatedly manipulated by the experimenter for a couple days and left to freely explore the Mobile HomeCage (MHC). From day 3, the animals were habituated to head fixation in the MHC by gradually increasing the time of each session, from 5 min initially, rising to 60 on day 6 (the day of the imaging session). The critical experimental design time points are listed in Figure 1(iv).

### 2.4 The multi-array probe

A 15 MHz multi-array probe was developed consisting in four compact linear arrays of 64 elements, with a pitch of 110 µm (IcoPrime-4D Multi-array, Iconeus, Paris, France). The high sensitivity of this probe is due to several factors, including the large active surface of each element (1.5 mm width), the small pitch (~λ), and the presence of an acoustic lens under each array enabling the acoustic energy to be focused on a slice of approximately 500 microns (minimum thickness at 8 mm depth) similarly to linear arrays. The four independent linear arrays were designed to be tightly assembled with only 2.1 mm from each other to minimize acoustical cross-talk (see Supplementary Fig. 3) and optimize the field of view. The total number of elements can be addressed simultaneously with a 256-channel scanner such as the Iconeus One system (256 channels).

The dedicated imaging sequence and the live Doppler reconstruction procedure were implemented in a live acquisition software (IcoScan, Iconeus, Paris, France), as described in the following sub-sections.

#### 2.4.1 Imaging sequence and beamforming with the multi-array probe

The imaging sequence was implemented using the same ultrafast plane wave transmission and reception scheme replicated for each of the four linear arrays. Four images (one per array) were simultaneously obtained from 4 × 200 compounded frames acquired at 500 Hz ($T_{integration}=0.4s$) using 4 × 8 tilted plane waves acquired at a pulse repetition frequency of 4 kHz (-12°, -8.57°, -5.14°, -1.71°, 1.71°, 5.14°, 8.57°, 12°). To compensate for the limited lateral aperture size (64 elements compared to 128 in conventional linear arrays), we used a trapezoidal beamforming grid with $\theta_{max}=12{}^{\circ}$, allowing the field of view to be extended on both sides and enabling the retrieval of deeper lateral brain regions.

#### 2.4.2 Clutter filtering

Each block of 0.4 s was filtered using a Singular Value Decomposition (SVD) clutter filter to separate tissue signal from blood signal and form a PD image (Demene et al., 2015).

Briefly, the signal matrix from a Doppler block $S\left(N_{x},N_{y},N_{z},N_{t}\right)$ was first reshaped to a Casorati matrix $M_{c}\left(N_{x}.N_{y}.N_{z},N_{t}\right)$ and decomposed by an SVD procedure as follows:

$$\begin{array}{c} M_{c}=U.S.V^{*} \end{array}$$ (1)

where S is a diagonal matrix with coefficients $\lambda_{i}$, corresponding to the ordered singular values associated with a spatial singular vector $U_{i}$ whose temporal variations are described by the temporal singular vector $V_{i}$. The signal associated with blood can be expressed as:

$$S_{blood}=\sum_{N_{cut}+1}^{200}U_{i\cdot }{\text{λ}}_{i\cdot }V_{i}^{*}$$ (2)

as the first $N_{cut}$ spatiotemporal modes are associated with tissue with high energy. For anesthetized experiments (as there isn’t any motion), we used a fixed threshold $N_{cut}=60$ (Demene et al., 2015). For awake experiments (episodic motion), the threshold $N_{cut}$ was set adaptively for each Doppler block using a fixed energy threshold (Baranger et al., 2018; Maresca et al., 2018).

#### 2.4.3 Motorized fast scanning with the multi-array probe

**Fig. 2.**
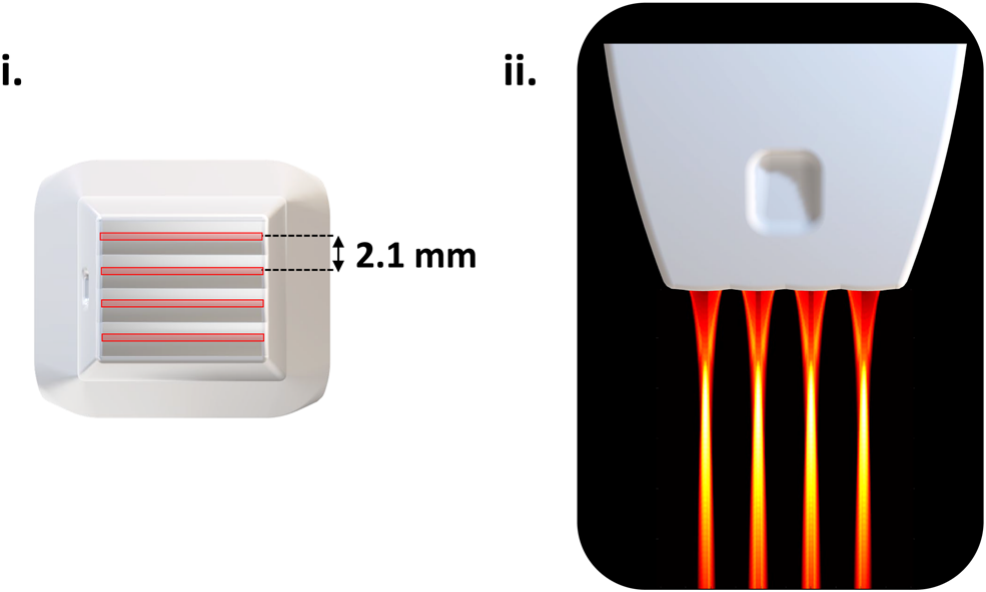
Multi-array probe. (i) Top view of the multi-array probe. The schematic of the probe head shows four acoustic lenses, separated by 2.1 mm. The slice thickness (0.5 mm) is represented with the red rectangles in the center of each array. (ii) Lateral view of head of the multi-array probe. The emitted pressure field, simulated with Field II software, is represented under each array, and thresholded at -6 dB.

**Fig. 3.**
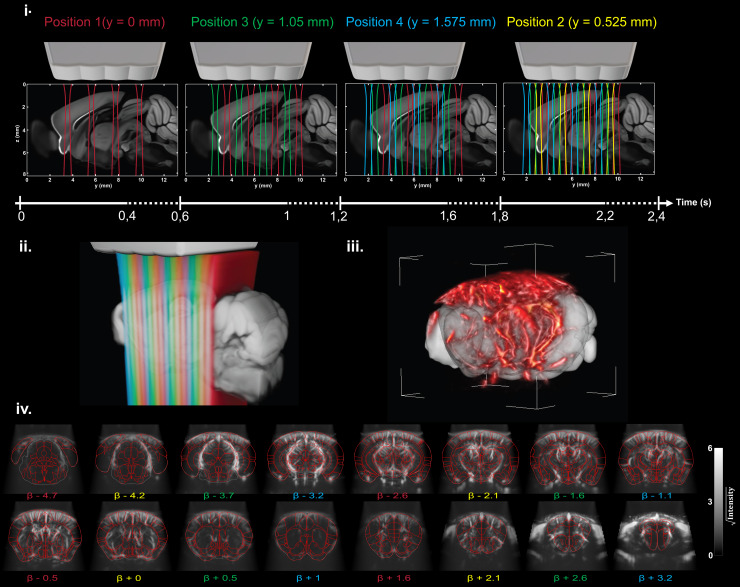
Principle of motorized fast scanning for whole-brain transcranial fUS imaging. (i) The probe is translated at each position following the order 1-3-4-2 (with interleaving) to minimize the translation distance and keep the translation time below 0.2 s. The step between slices is set to 0.525 mm, allowing homogeneous scanning of the dead volume between two arrays (2.1 mm). At each position, the probe rests for 0.4 s to acquire 4 × 200 compounded frames at 500 Hz. The resulting pressure field (simulated with Field II (Jensen, 1997)) is represented under each array, and overlaid on the two-photon Allen template. Every 2.4 s, the 16 continuous slices (ii) are beamformed, processed, and concatenated to form a 3D volume (iii). These 16 PD slices are also depicted in (iv) with their corresponding anatomical coordinate relative to Bregma coordinates (in mm) and overlaid with the envelope of the main Allen atlas regions. This cycle is repeated constantly during the whole acquisition. The PD scan represented in (iv) was performed on an anesthetized mouse.

As illustrated in Figure 2(i), the four arrays are separated by 2.1 mm, and the acoustic lenses allow four acoustic beams to focus along the elevation direction with a full-width half-maximum (FWHM) of 0.5 mm. By translating the probe at four positions separated by 0.525 mm (a quarter of the inter-array distance, Fig. 3(i) and (ii)), we can achieve a final volume of 16 contiguous slices with a repetition time (TR) of 2.4 s (Fig. 3(iii) and (iv)). Up to a step of 0.525 mm, the scanning is considered non-interrupted slicing, ensuring that the whole-brain volume is sampled without any gaps between the slices. The TR of 2.4 s not only considers the integration time ($T_{integration}=0.4\;s$), but also considers the dead time during which the probe is translated ($T_{translation}=0.2\;s$) and the number of positions (four). To avoid long displacements and make sure that $T_{translation}$ does not last more than 200 ms between the last position and the first one (before starting each new volume), the scanning order of the different positions was optimized to limit the maximum displacement to two steps (1.050 mm) using an interleaved sequence (1-3-4-2 if $n_{positions}=4$). Regarding this maximum displacement and considering the configuration of our motor setup, the maximal translation time was estimated at 0.1 s. All sequence parameters are summarized in Table 1, and the whole procedure is described in Figure 3.

**Table 1.** Summary of the recommended sequence parameters for whole-brain SMS fUS imaging.

Spatial specifications					Temporal specifications			
Field of view	Depth	In-plane resolution	Number of slices	Step between slices	Frame rate	Doppler integration	Pause duration	Repetition time
β-4.9 to β+3	1 mm to 11 mm	100 µm × 100 µm	16	525 µm	500 Hz	0.4 s	0.2 s	2.4 s

If $n_{positions}>1,\text{TR}$ can be expressed as:

$$\begin{array}{c} TR=n_{positions}\left(T_{integration}+T_{translation}\right) \end{array}$$ (4)

$n_{positions}$ and $T_{integration}$ parameters can be set to different values, and we will discuss the implications of temporal resolution and SNR later in the Section 4.

#### 2.4.4 Comparison with RCA and matrix transducers

We performed acquisitions of 180 s with the MUX-FPM, the RCA, and the multi-array probes on the same animal, before and after craniotomy (removal of the skull) and compared the image quality.

For the surgery and the following brain imaging session, the mouse was anesthetized with an initial intraperitoneal injection of ketamine and xylazine mixture. Then, anesthesia was maintained with a 1.5% isoflurane supply and the animal physiology was monitored following the protocol already described in Section 2.2.1. An additional subcutaneous injection of buprenorphine (0.1 mg/kg) was administered to provide analgesia, A 1 × 1 cm skull window was removed by drilling (Foredom) at low speed using a microdrill steel burr (Burr number 19007-07, Fine Science Tools) while leaving the dura intact.

The imaging sequence parameters for the RCA and MUX-FPM are detailed in Supplementary Table 1. All the probes were centered at 15 MHz.

For the RCA sequence, 20 tilted plane-wave were transmitted alternatively with the rows and the columns, while backscattered echoes were always received with the orthogonal aperture. We used the XDoppler approach (Bertolo, Sauvage, et al., 2021) to obtain an isotropic PSF with reduced side-lobe levels. The MUX-FPM sequence was designed to drive one sub-aperture of 32 × 8 piezo elements (representing a quarter of the whole aperture) at a time, and the back-scattered echoes were received on the whole aperture after four transmissions and receptions, thus allowing a frame rate of 333 Hz for nine tilted plane waves. The MUX FPM and RCA volumes were further averaged over 2.4 s to match the volume rate of the multi-array acquisition scheme.

All volumes were registered and resampled in the same space to allow the extraction of PD intensity profiles along the same voxels, and Contrast to Noise Ratio (CNR) estimation from the same vessel and background regions of interest for the different acquisitions.

### 2.5 Automatic atlas registration, and ROI segmentation

All volumetric scans were co-registered to an average scan and aligned to a standard Doppler reference template, already pre-aligned with the Allen Mouse Brain Atlas common coordinates framework (Nouhoum et al., 2021; Wang et al., 2020) (Fig. 3(iii)). An initial affine transformation matrix was first determined using a convolutional neuronal network (Blons et al., 2022) trained to identify nine vascular landmark locations in an angiographic scan. From the identification of these landmarks in both the scan and the reference template, we could estimate a first affine transformation aligning the scan to the Doppler reference. A refined affine transformation was finally determined from this initial transformation by running an iterative intensity-based registration algorithm (Nouhoum et al., 2021).

An indirect evaluation of the registration was proposed by introducing a “matching score”. This score is defined as the average correlation between the registered scan and the averaged template from the whole dataset. This score is reported for each session in Supplementary Figure 1.

### 2.6 Scans pre-processing pipeline

**Fig. 4.**
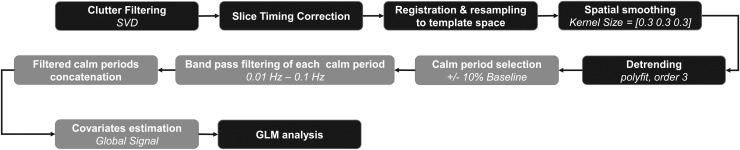
Block diagram describing each step of the processing pipeline. Steps represented in gray blocks are specific to awake resting-state data pre-processing.

The entire pre-processing pipeline is illustrated in Figure 4, and further details about every step are provided in this section. Slice timing correction (STC) was systematically applied by resampling the data on a common time basis (first position) to consider the delays in slices, using linear interpolation.

**Fig. 5.**
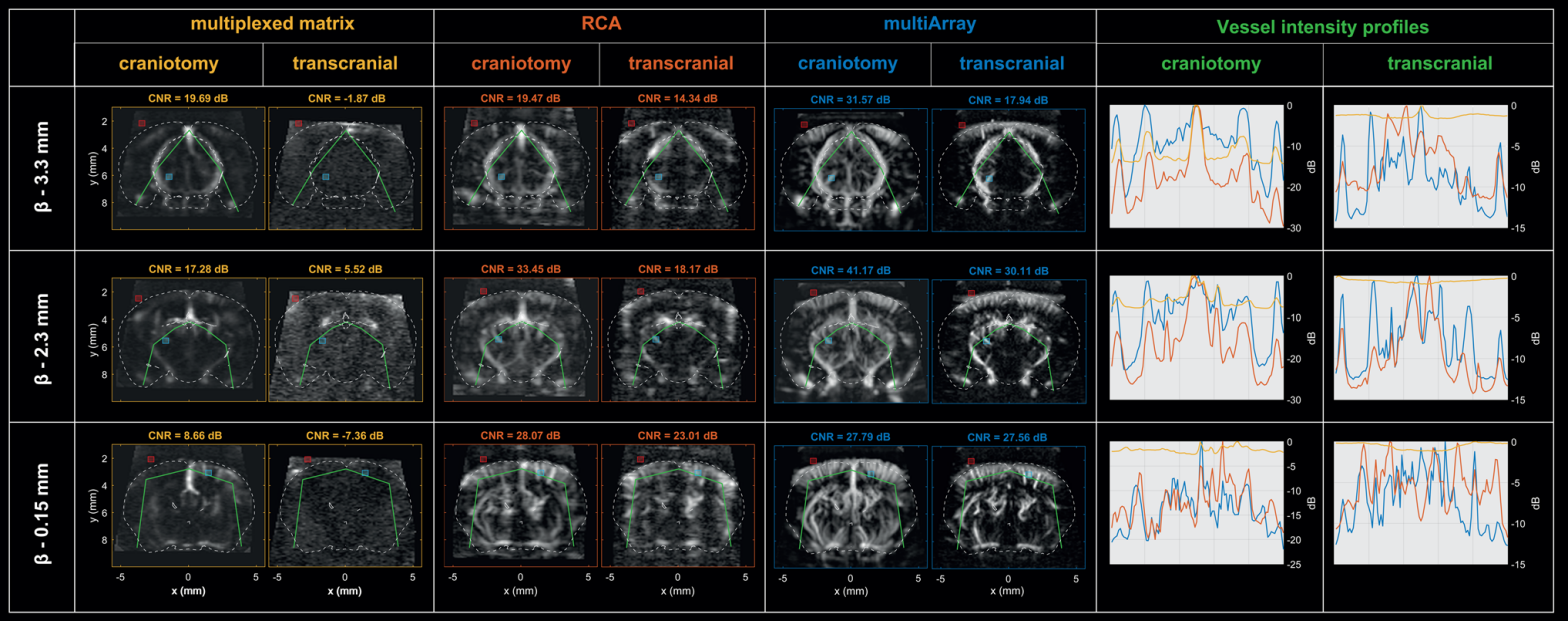
Assessing the imaging quality before and after craniotomy: comparative analysis of the multi-array probe against RCA and MUX-FPM probes. Comparisons were focused on three coronal slices, represented in each row of this comparative table. For each probe, the first column represents PD images after craniotomy whereas the second one represents PD images before craniotomy. For each slice, the vessel ROI is represented with the cyan square whereas the background ROI is represented with the red square. The green line indicates the voxels along which PD profiles were extracted for contrast comparison (last column). Whereas the multi-array probe provides the best imaging quality before and after craniotomy, the MUX-FPM is not sensitive enough to detect blood flow through the skull.

Then, the baseline low-frequency drift was estimated (voxel-wise) with *polyfit* function (MATLAB), using a polynomial of degree 3, and subtracted from the fUS signal.

Despite adaptative clutter filtering, a variable portion of certain acquisitions had to be removed in each scan because of the high motion. Calm “resting” periods were automatically determined from the whole-brain global signal (GS) Doppler profile. The baseline of the GS was first estimated with a linear regression. Then, periods were considered as calm when the GS standard variations were kept below 5% of the GS baseline level for at least 60 s, and a calm score was computed as the total useful duration corresponding to resting periods during a whole acquisition. This score is reported in Supplementary Figure 1. Two acquisitions with less than 10 min of calm periods were removed from the resting-state analysis. For the corresponding animals, another imaging session led to less artefactual acquisitions, and was thus considered in the FC analysis.

To study the slow CBV fluctuations associated with resting-state FC, the fUS signal of each calm period was first standardized before applying a bandpass filter. According to the fMRI literature (Biswal et al., 1995) and recent fUS studies (Nunez-Elizalde et al., 2022) characterizing these low-frequency oscillations, the frequency band was chosen between 0.01 Hz and 0.1 Hz. Then, all the filtered calm periods were temporally concatenated. Finally, the GS of the resulting pre-processed scan was derived and considered as a confounding variable to reduce non-neuronal sources of variance for awake resting-state FC data. The choice to integrate the global signal regression (GSR) procedure for the processing of our awake dataset will be discussed later in the manuscript.

### 2.7 Statistical analysis

#### 2.7.1 General linear model (GLM) for visual stimulation activation maps and resting-state FC seed-based maps estimation

Activation maps (visual stimulation) and seed-based maps (resting-state FC) were computed using a GLM applied on pre-processed scans.

For visual stimulation, the stimulus response was modeled by convolving the stimulus pattern with a four half-cosine canonical hemodynamic response function. This signal was rescaled so that its value was zero when there was no stimulus and one during the stimulus. The baseline could then be directly extracted from our model from the intercept value.

For seed-based maps, the expected signal was taken as the average CBV time course in the seed ROI. A t-statistic with its corresponding p-value was derived for each voxel to assess the GLM significance, comparing the baseline condition (contrast = 0) and the stimulus (contrast = 1).

The familywise error rate (FWER) for subject-level analysis was controlled by the Bonferroni procedure, adopting a false discovery rate of 0.05.

Group-level significance was assessed with a one-sample Student t-test performed on the individual t-maps, with a false discovery rate of 0.05. Correction for multiple comparisons was done with maximal statistic permutation testing combined with threshold free cluster enhancement (TFCE) (Smith & Nichols, 2009).

#### 2.7.2 Average relative CBV (rCBV) profiles in activated areas during visual stimulation experiments

Hemodynamic responses to visual stimulation were estimated by computing the relative change in CBV (rCBV) in percentage, obtained by subtracting the CBV baseline and dividing by the CBV baseline afterwards.

**Fig. 6.**
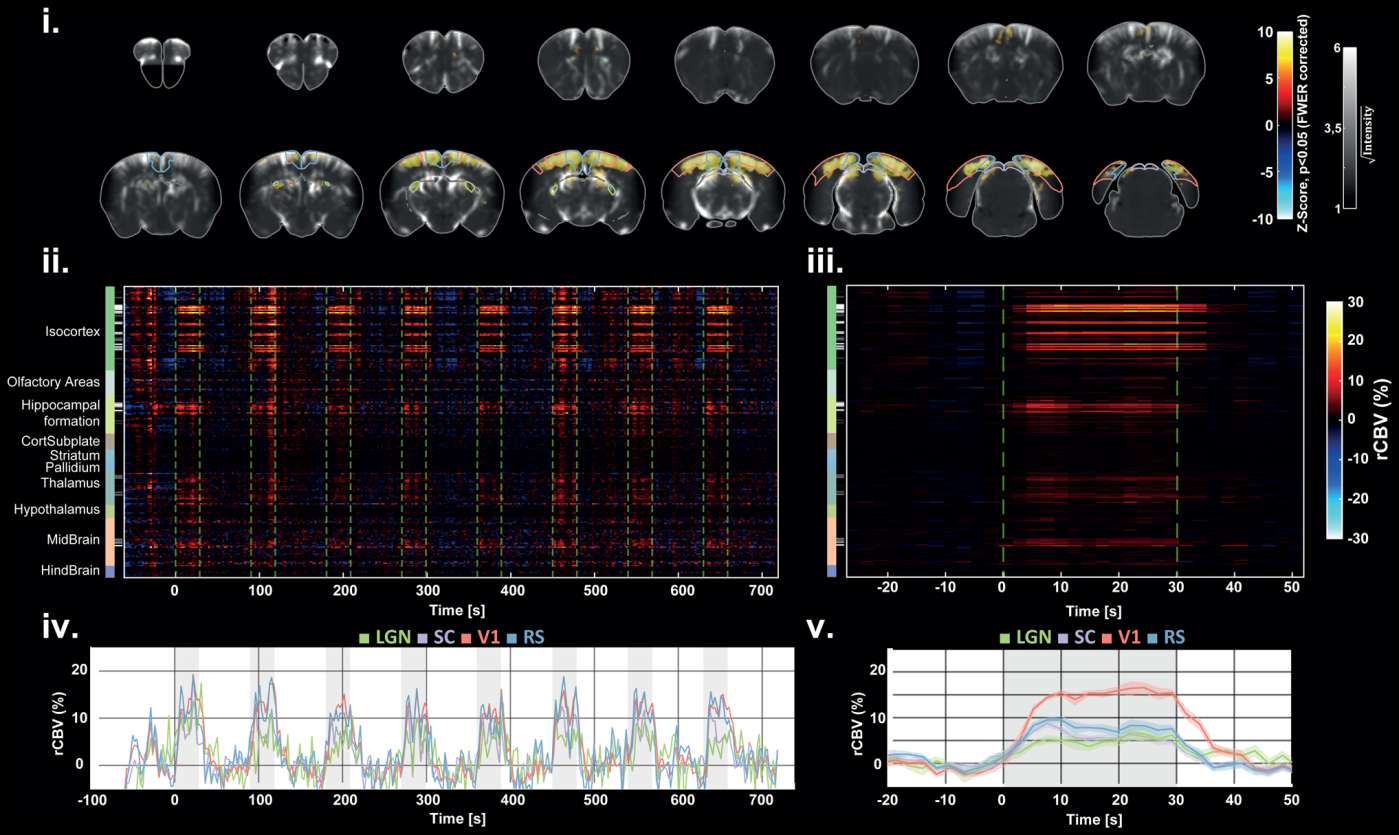
Subject-level response after visual stimulation. (i) Subject-level activation map (p < 0.05, FWER corrected with Bonferroni procedure) overlaid with the PD angiography from the most anterior (top left) to the most posterior acquired slice (bottom right), reveals significant activation in major brain areas of the visual system: contours for the V1, RS, SC, and LGN regions are depicted on each slice. (ii) Raster plot of rCBV time course extracted in 228 Allen regions covering the whole brain. The gray-scale colorbar indicates the percentage of activated voxels in each region (black = 0%, white = 100 %). Green dashed lines indicate the beginning and the end of each stimulus. (iii) Cross-trial averaged raster plot. (iv) rCBV curves extracted in V1, RS, SC, and LGN, showing single-trial detections at each stimulus. (v) The average rCBV curves (cross-trial) show an increase of the rCBV from 5% for the LGN to 15% for V1 during the ON-time.

The rCBV time profiles were extracted in 228 Allen regions of interest (Supplementary Table 2) and represented through temporal raster plots (Fig. 6(ii)). The percentage of significantly activated voxels in each of these regions was determined by overlapping the Allen segmentation to the p-value map obtained with the GLM analysis (gray-scale colorbars on raster plots). We also plotted the rCBV of significantly activated voxels in important regions of the visual pathway (Fig. 6(iv)).

**Fig. 7.**
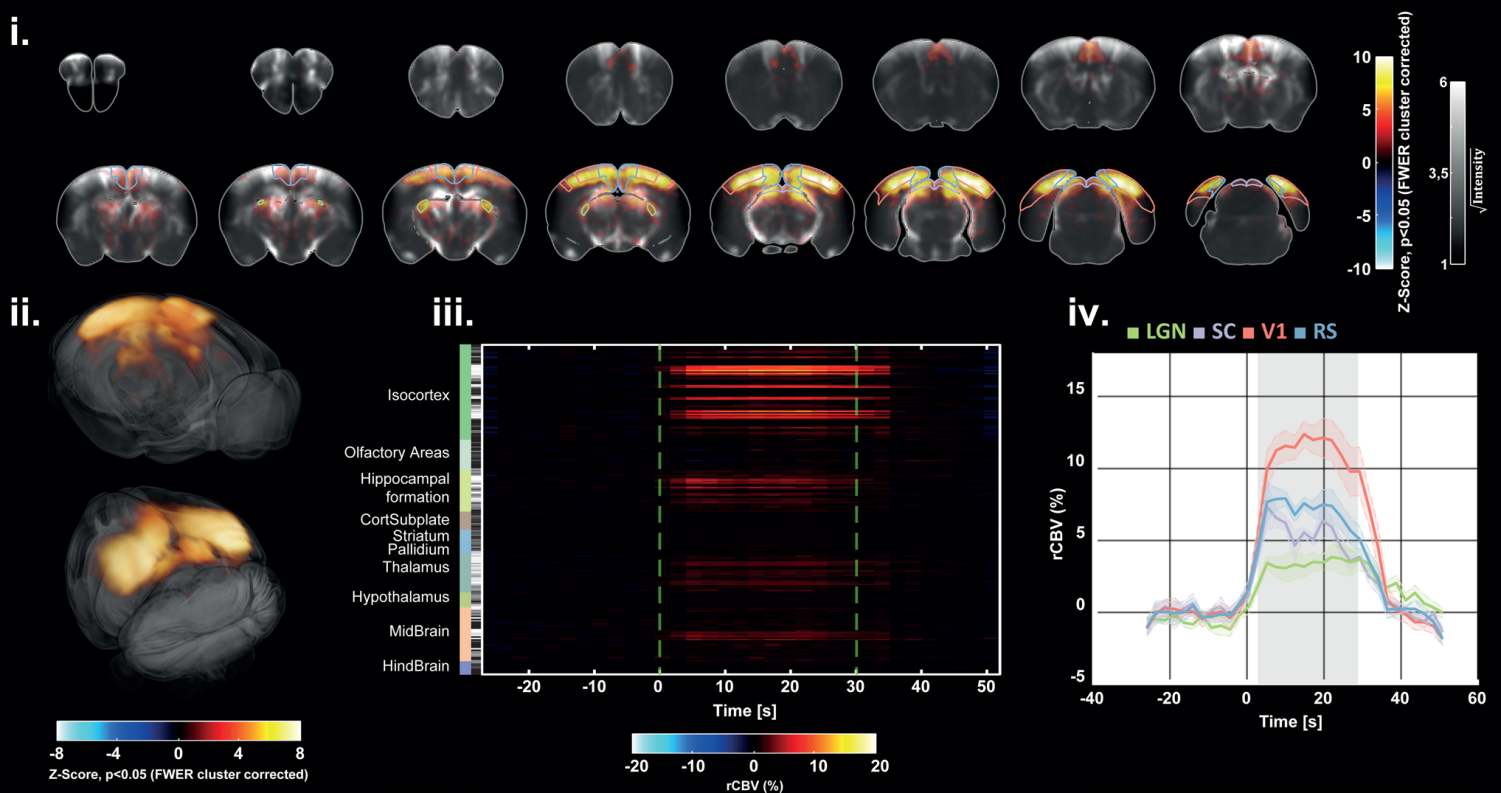
Group-level response after visual stimulation (n = 6). (i) Average activation map (two-tailed t-test, p < 0.05, FWE corrected using TFCE and maximal statistic permutation testing), overlaid with the average PD angiography (cross-subject), from the most anterior slice (top left) to the most posterior slice (bottom right). (ii) 3D renderings (Amira software) of the average activation map, thresholded with significant voxels. (iii) Raster plot of averaged rCBV profiles (cross-trial and cross-subjects) extracted in 228 Allen regions. The gray color bar indicates the percentage of significant voxels in each region (black = 0%, white = 100 %). (iv) Average rCBV (cross-trial and cross-subjects) profiles extracted from important regions of the visual pathway show an increase of the rCBV going from 4% for the LGN to 12% for V1 during the ON-time.

For each subject, the inter-trial rCBV was estimated by averaging the eight trials within an imaging session (Fig. 6(ii, v)). Finally, the inter-subject (n = 6) and inter-trial rCBV was finally derived by averaging inter-trial rCBV profiles of each subject (Fig. 7(iii, iv)).

#### 2.7.3 Functional connectivity matrix estimation

Thanks to the registration procedure, the Allen atlas segmentation could be used to perform automatic Allen-based CBV extraction over more than 200 brain regions of interest (ROI) distributed in the whole brain (listed in Supplementary Table 2). The Pearson correlation coefficient was computed between every pre-processed and spatially averaged signal (in each ROI). Subject-level FC matrices were Fisher-transformed and averaged across subjects (n = 6). The group-average matrix was finally re-transformed to Pearson correlations. We also performed a one-sample t-test (two-tailed, p < 0.05, FDR corrected) to test whether the average coefficients were different from zero.

For each session, the value of the correlation between two symmetric regions taken in the somatosensory cortex (SS), and between the somatosensory cortex and the anterior cingulate cortex (ACA) was taken as a quality control metric, describing the specific or unspecific character of functional connectivity (Supplementary Fig. 1) (Grandjean et al., 2017).

#### 2.7.4 Independent component analysis (ICA) for functional networks identification

Independent components (ICs) were estimated by running *fastICA* 100 times with *icasso* stabilization MATLAB algorithms (Himberg & Hyvarinen, 2003; Ntekouli, 2019) on co-registered pre-processed and temporally concatenated resting-state FC scans (awake condition, n = 6). Different dimensionalities were tested: 15, 25, and 35. We chose to set the number of ICs to 25, as it was found to best represent the heterogeneity of our dataset with a good stability. Finally, plausible FC networks were classified manually by following the same rules as the ones proposed by Zerbi *et al.* (2015) and Grandjean *et al.* (2017) fMRI studies. The general assumption behind this identification is: if there are active functional networks, those functional network components will closely match known structural networks among vascular or noise components. Out of the 25 group-level ICs, we identified 16 spatial components associated with functional systems. To enhance functional regions visualization, each spatial map was scaled to Z-scores and thresholded to |Z|>3 (corresponding to p < 0.001). Finally, volumetric representations were constructed by extracting the boundary surface of the resulting binarized mask.

## 3 Results

### 3.1 The multi-array probe is more sensitive than RCA and matrix arrays

The results of the image quality comparison between the MUX-FPM, the RCA and the multi-array probes are presented in Figure 5. We focused our comparisons on three coronal slices, intersecting β-3.3 mm, β-2.3 mm, and β + 0.15 mm. The sensitivity was assessed by comparing CNR estimations from the same vascular and background regions for all acquisitions. Vessel’s intensity profiles were also extracted along the same voxels.

In both trepanned and transcranial conditions, the multi-array provides a better image quality than MUX-FPM and RCA probes, with higher CNR values. Higher peak amplitude and more small vessels were revealed with PD intensity profiles for the multi-array.

In transcranial condition, the vascular signal is completely lost with the MUX-FPM, and the multi-array offers a better contrast compared to the RCA on the vessel’s intensity profiles.

Interestingly, the CNR obtained with the multi-array in transcranial condition is still better than the one measured with the MUX-FPM without the skull.

### 3.2 Functional hyperaemia induced by visual stimulation in lightly anesthetized mice

To validate the multi-array approach, we first evaluated the ability to detect CBV responses in the whole brain during visual stimuli.

Figure 6 shows the visual responses obtained in one representative mouse after one experiment of 780 s. Activation maps obtained with GLM analysis revealed significant activation in all regions of the visual pathway: the lateral geniculate nucleus (LGN), the superior colliculus (SC), the primary visual cortex (V1), and the retrosplenial cortex (RS), as represented in Figure 6(i). The raster plot in Figure 6(ii) shows the rCBV response in the whole brain. The percentage of activated voxels was derived in each of the 228 segmented Allen regions (Supplementary Table 2) and is represented with the gray colorbar. Moreover, the rCBV time profile shows single-trial detection in each region. On average (across trials), the rCBV increase reached 5% in the LGN, 7% in the SC, 16% in the V1, and 9% in RS in this experiment.

A second-level statistical analysis was then performed on a population of six subjects. Voxels with significant activation are represented on coronal slices (Fig. 7(i)) and in 3D renderings (Fig. 7(ii)). Significant activation was detected in the Thalamus, in the Midbrain, in the Hippocampal formation, and in the Isocortex. The percentage of activation in each of the 228 Allen regions (gray colorbar in Fig. 7(iii)) is reported in Supplementary Table 2. On average (cross-trials and subjects), the rCBV increase reached 4% in the LGN, 6% in the SC, 7.5% in the RS, and 12% in the V1 during the ON-time (Fig. 7(iv)).

### 3.3 Resting-state functional connectivity in awake mice

To study the dataset associated with resting-state functional connectivity in awake conditions (n = 6), we performed different statistical analyses. First, we performed a seed-based analysis, then we derived the average functional connectivity matrix, and we finally investigated spatial ICA to identify functional networks.

**Fig. 8.**
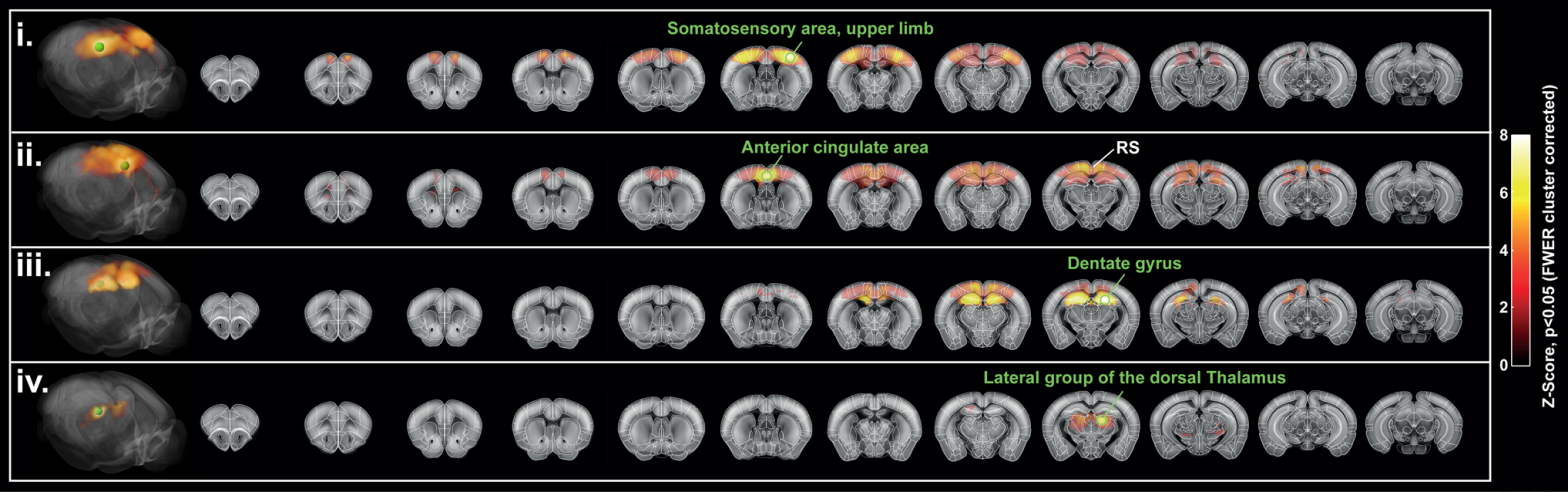
Seed-based analysis reveals long-range FC patterns (n = 6). Each row represents an average seed-based map across the awake dataset (n = 6), thresholded with significant connectivity (one-tailed t test), p < 0.05, FWER cluster corrected using TFCE. Seed regions are denoted by the green legends: (i) somatosensory area, upper limb, (ii) Anterior cingulate area, (iii) Dentate gyrus, (iv) Lateral group of the dorsal Thalamus. Maps were resampled in the Allen mouse template. Volume renderings (left) are performed with Amira software. Activation maps are also represented on coronal slices overlaid with the two-photon Allen mouse template.

The seed-based analysis revealed significant and long-range functional connectivity patterns across the awake dataset. When the seed was placed in the upper limb region of the primary somatosensory area (SSp-ul), strong FC was measured in the contralateral region, and in other regions of the primary somatosensory cortex, such as the trunk (SSp-t), lower limbs (SSp-ll), the secondary motor cortex (MOs), and the hippocampal region (HPC, Fig. 8(i)). The specific midline associative cortices hub involved in the default mode network (DMN) previously described in the fUS (Grandjean et al., 2017) and fMRI (Gozzi & Schwarz, 2016; Grandjean et al., 2017; Sforazzini et al., 2014) literature was then identified by placing the seed in the dorsal part of the anterior cingulate area (ACA, Fig. 8(ii)). Finally, sub-cortical inter-hemispheric FC was also found in both hippocampal and thalamic regions when placing the seed in the dentate gyrus or in the lateral group of the dorsal thalamus, respectively (Fig. 8(iii)). The map obtained with the DG seed also revealed hippocampo-cortical connections. These patterns were also identified at the subject level, as demonstrated in Supplementary Figure 2.

**Fig. 9.**
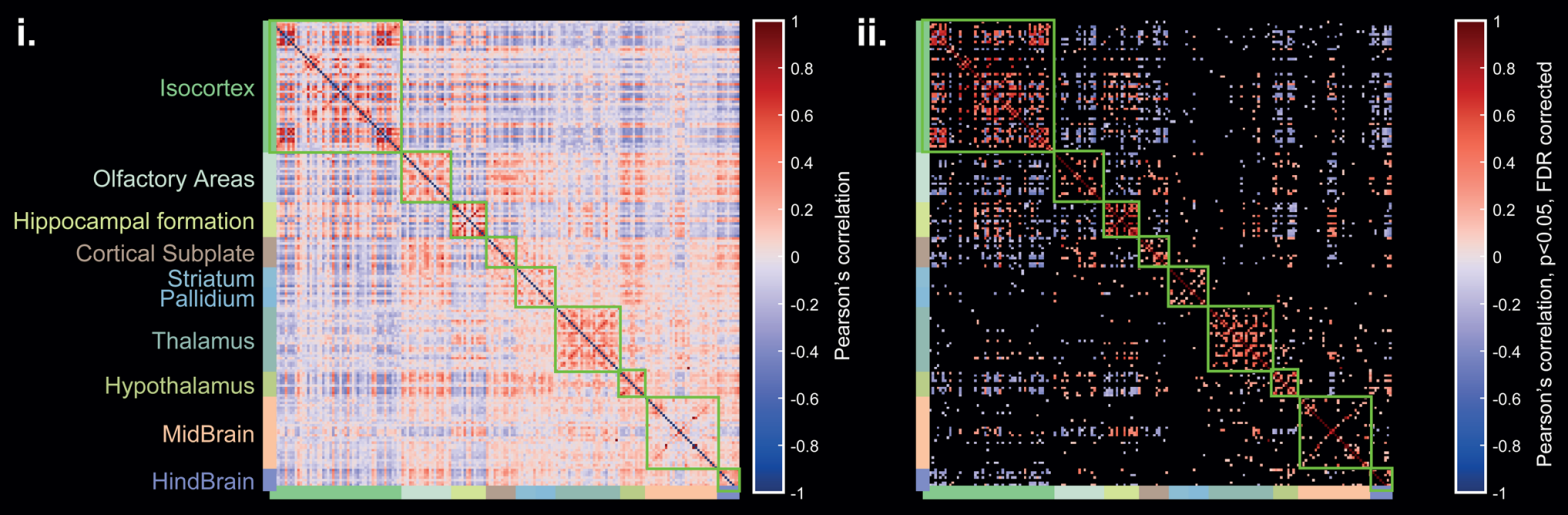
Average functional connectivity matrix (n = 6) derived for more than 200 Allen cortical and subcortical structures which are listed in Supplementary Table 2 (i). Strong inter-hemispheric FC (anti-diagonals in green squares) is measured in both cortical and sub-cortical regions. Significant coefficients (one-sample t-test, p < 0.05, FDR corrected) are represented on the right matrix (ii).

Then, the average functional connectivity matrix provided a global view of the whole brain FC (Fig. 9). The Allen-based and mirrored (left/right) segmentation of ROIs allowed different FC patterns in each major brain region to be exposed. The strongest inter-hemispheric correlation coefficients were observed in the isocortex, the hippocampal formation, the olfactory areas (OA), the thalamus (TH), and the hypothalamus (HT). Interestingly, the OA region was found to be highly functionally connected with different sub-cortical structural regions such as the cortical subplate (CS), the striatum (ST), and the HT, in good agreement with the results shown in a recent fMRI study in awake mice (Gutierrez-Barragan et al., 2022).

**Fig. 10.**
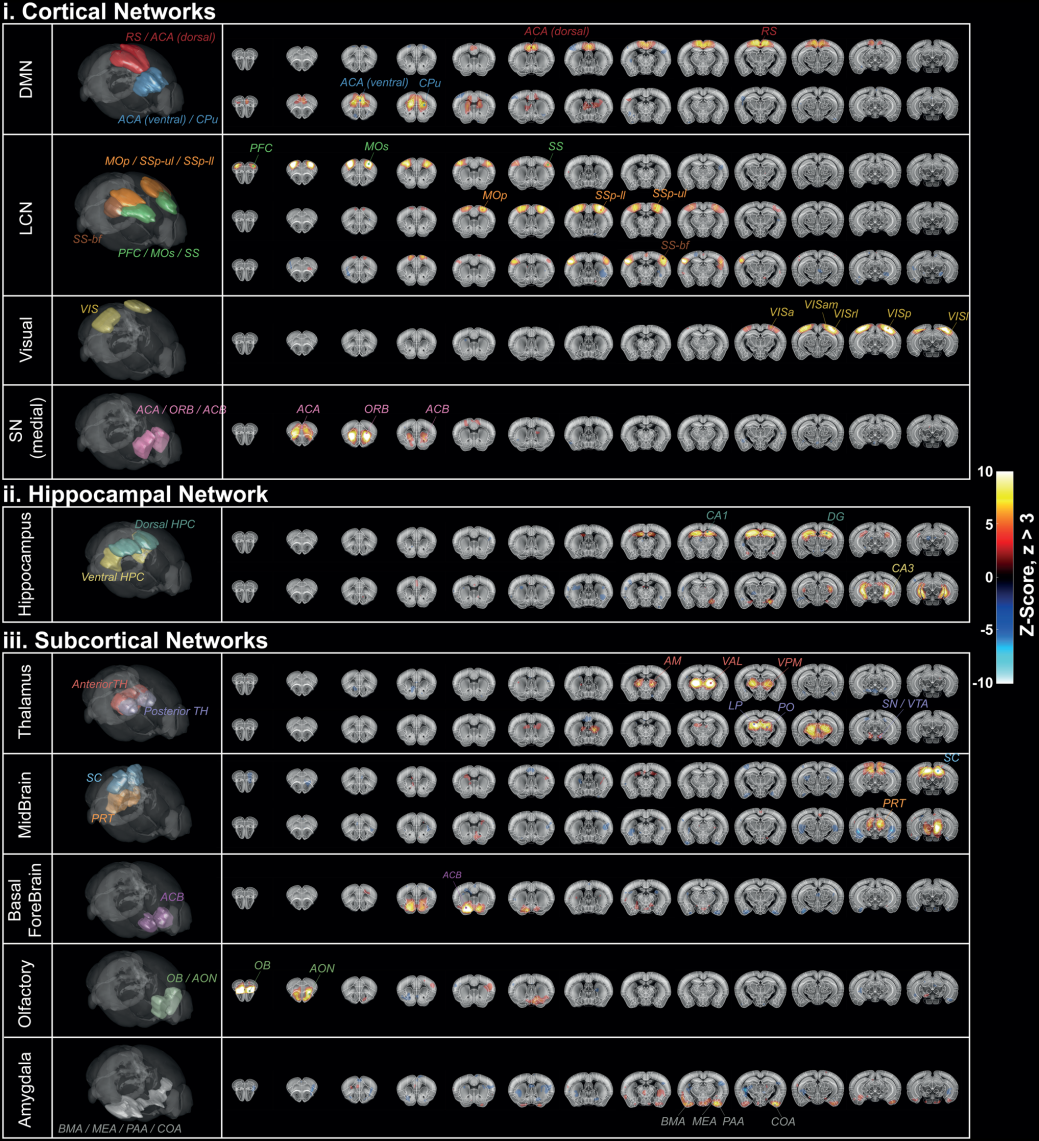
Functional networks identified with ICA. Identified networks were classified in three different groups (cortical (i), hippocampal (ii), and sub-cortical (iii)). Each network (labeled in the first column and represented in 3D in the second column) comprises at least one independent component. For each component, abbreviations of overlapped structural regions are captioned. In the last column, coronal sections of spatial maps are overlaid with the two-photon Allen template. RS = retrosplenial area, ACA = anterior cingulate area, MOp = primary motor area, SSp = primary somatosensory, ul = upper limb, ll = lower limb, bf = barrel field, PFC = prefrontal cortex, Mos = secondary motor cortex, VISa = anterior visual area, VISam = anteromedial visual area, VISrl = rostrolateral visual area, VISp = primary visual area, VISl = lateral visual area, ORB = orbital cortex, ACB = nucleus accumbens, HPC = hippocampus, CA = cornu ammonis, DG = dentate gyrus, AM = anteromedial nucleus, VAL = ventral anterior-lateral complex of the thalamus, VPM = ventral posteromedial nucleus, LP = lateral posterior nucleus of the thalamus, PO = posterior complex of the thalamus, SN = substantia nigra, VTA = ventral tegmental area, SC = superior colliculus, PRT = pretectal region, ACB = nucleus accumbens, OB = olfactory bulb, AON = anterior olfactory nucleus, BMA = basomedial amygdalar nucleus, medial amygdalar nucleus, PAA = Piriform-amygdalar area, COA = cortical amygdalar area.

Finally, group-ICA revealed the presence of 16 resting-state functional networks. Five of them were classified as cortical networks (Fig. 10(i)), one as a hippocampal network (Fig. 10(ii)), and five as sub-cortical networks (Fig. 10(iii)). These networks span various brain regions defined by the Allen brain atlas and correlate with the brain structural connectivity with a systematic bilateral organization. Resting-state networks previously described in the fMRI literature were identified in our volumetric fUS data, involving the same anatomical regions. The identification of the DMN, the latero-cortical network (LCN), and the medial part of the salience network (SN) is in good agreement with the triple-network organization for the mouse brain previously proposed (Mandino et al., 2022). For the SN, the absence of activation in the agranular insular is argued in the discussion part. The large field of view allowed the complete mapping of the visual network. Deeper networks were also characterized, including the hippocampus (HPC) and several sub-cortical networks, namely the thalamus (TH), the midbrain (MB), the basal forebrain (BF), the olfactory (OF) and the amygdala (Amy) networks. The presence of BF and OF networks is in good agreement with previous fMRI observations, supporting that high functional activity in these arousal regions is related to conscious wakefulness (Gutierrez-Barragan et al., 2022). Several networks were described with more than one IC. The DMN was decomposed into a first component connecting the ACA and the RS together and a second one that was more specific to anterior ACA, as was previously observed in fMRI studies (Grandjean et al., 2017; Zerbi et al., 2015). The LCN was associated with three different components. The more anterior one was found to connect the prefrontal cortex (PFC), the secondary motor cortex (MOs), and the somatosensory cortex (SS). Among the two anterior components, a first one involved the primary motor cortex (MOp), the upper-limb and lower-limb regions of the primary SS cortex (SSp-ul and SSp-ll) whereas the more lateral one matched the barrel-field cortex (SS-bf). HPC and MB networks were described by two dorsal/ventral submodules, and the TH network was decomposed into two lateral/ventral submodules, matching with different sub-cortical nuclei specified in Figure 10(iii).

The elevation extent of each network is reported in Supplementary Table 3.

## 4 Discussion

Compared to other preclinical neuroimaging modalities, fUS imaging benefits from several advantages such as a high spatial resolution, a high sampling rate, and a full-depth, non-invasive penetration into the mice brain. However, monitoring resting-state functional connectivity (rsFC) with brain-wide coverage in mice with an intact skull had, up to now, never been performed because of sensitivity and volume-rate issues (15-17, 35). In this study, we present the use of a new simultaneous multi-slice (SMS) approach based on a multi-array probe to perform transcranial brain-wide fUS imaging of functional activity and connectivity in anesthetized and awake mice. This new method allows for an acceleration in data acquisition equal to the number of simultaneously insonified slices (four), without compromising the sensitivity when compared to classic linear ultrasound arrays.

The sensitivity of our approach was highlighted by the comparison of the imaging quality with other methods in the same animal. Compared to the images acquired with the RCA and MUX-FPM probes, the multi-array images provided higher CNR values and better contrasts on vessel intensity profiles, in both trepanned and transcranial conditions.

Our hybrid approach using a multi-linear array allows for functional connectivity imaging over 16 complete coronal slices, providing a more comprehensive view of brain functional networks in the whole mouse brain through the skull.

We first conducted SMS-fUS acquisitions to investigate task-based functional activation and obtained reliable hyperemia in different brain regions of the visual system in response to light stimuli in lightly anesthetized mice. Notably, we found statistically significant activations in the lateral geniculate nuclei, the superior colliculi, the primary visual cortex, and, more surprisingly, within the retrosplenial area at both the subject level and group level. While the retrosplenial cortex is not typically found to be activated following visual stimulation in fMRI studies (Dinh et al., 2021; Niranjan et al., 2016), this brain region has been shown to exhibit visual responses using GCaMP imaging techniques (Murakami et al., 2015), and has been included in an extended retinotopic organization of the mouse brain (Zhuang et al., 2017). Consistently, significant hyperemia has already been demonstrated in the retrosplenial area using fUS in awake head-fixed mice, independently of stimulus directions or the presence of an optokinetic reflex (Macé et al., 2018). Taken together, these observations further support the high sensitivity of fUS imaging and its ability to faithfully report neuronal activity. In this study, we used a simple flashing LED stimulus, but more complex visual tasks could be performed using the same approach such as multi-directional drifting grating stimuli (Macé et al., 2018). Similarly, this paradigm could be straightforwardly transposed to the mapping of the brain correlates of complex behavior or cognitive tasks in awake mice in future studies.

Resting-state functional connectivity was then evaluated in awake head-fixed mice under stimulus-free conditions. Leveraging our previously published automatic registration to the Allen Mouse Brain Atlas (Nouhoum et al., 2021), we performed seed-based analysis, derived an average functional connectivity matrix over 200 segmented regions, and identified large-scale functional connections spanning several coronal slices. Expanding previous work using fMRI (Gutierrez-Barragan et al., 2022; Tsurugizawa et al., 2020), our work provides a fine-grained comprehensive description of the organization of mouse brain functional networks during wakefulness. Unsupervised multivariate ICA analysis has revealed 16 functional networks that cover the whole brain and that strikingly resemble those previously described in the fMRI literature. We found relevant inter-hemispheric connectivity within the cortical, hippocampal, basal ganglia, and sub-cortical regions at both subject and group levels. Based on previously published fMRI studies (Grandjean et al., 2017; Mandino et al., 2022; Zerbi et al., 2015), relevant components have been manually classified into major networks identified in the mouse brain: the default mode network (including midline and associative regions), the lateral cortical network (including somatosensory-motor executive areas), the visual network, as well as the salient network (including the most anterior part of the anterior cingulate and orbitofrontal cortices). Subcortical components included the hippocampal network, the thalamic network, as well as modes within the midbrain, the basal forebrain, the olfactory nucleus, and the amygdala. We found two independent hippocampal networks corresponding to the dorsal and ventral hippocampus, as described in the literature (Zerbi et al., 2015), as well as two independent thalamic networks corresponding to the anterior and posterior thalamus, less often described in the fMRI literature. Together, these observations attest to the high sensitivity of our approach through its ability to detect reliable spontaneous coactivations in the deepest regions of the mouse brain, even in the transcranial setting. More advanced analyses such as dynamic functional connectivity and coactivated patterns (CAPs) could help provide a more accurate description of the mouse functional connectome and facilitate comparisons with fMRI datasets.

Several limitations must be considered when interpreting the results. First, certain areas of the brain remain more difficult to image due to stronger skull aberrations, mostly linked to the skull shape or presence of sutures. This includes the cerebellum, the auditory cortex, temporal areas, and insular cortex. This explains the absence of significant functional connectivity in these regions. Applying aberration correction techniques to the ultrasound field may help recover signals in those areas. Second, the current field of view, which was specifically optimized for the adult mouse brain, would be too restricted for rats or larger animals. Future development of 512-channel scanners with higher computational power will enable the conceptualization of larger multi-arrays transducers to overcome this limitation. Alternatively, larger piezo elements with reduced central frequency could be considered to increase the probe aperture, but with a tradeoff on in-plane spatial resolution.

Moreover, the use of global signal regression (GSR) as a preprocessing step for resting-state signals is highly debated in the fMRI literature. GSR was used in the present study to reduce contributions from non-neuronal sources, notably motion artifacts, in awake resting-state signals (Rabut et al., 2020). However, as GSR has been shown to introduce spurious negative correlation values in FC networks (Murphy & Fox, 2017), great care should be taken when interpreting negatively correlated regions in seed-based maps and GLM analyses. Note that the preprocessing of task-evoked data from anesthetized mice did not require GSR as there were no motion artifacts.

Overall, our approach showed promising results with a TR of 2.4 s—a theoretical limit for resting-state functional connectivity (with respect to Nyquist-Shannon theorem and considering an upper frequency of 0.2 Hz for the RS bandwidth)—but it is still slower than what could be reached using a matrix or row-column array (Brunner et al., 2020; Rabut et al., 2019; Sauvage et al., 2019). In our study and others (Aydin et al., 2020; Bertolo, Nouhoum, et al., 2021; Boido et al., 2019; Ferrier et al., 2020; Gesnik et al., 2017; Macé et al., 2018; Nunez-Elizalde et al., 2022; B. F. Osmanski et al., 2014; B.-F. Osmanski et al., 2014; Rabut et al., 2020; Sieu et al., 2015; Tiran et al., 2017), high sensitivity Doppler frames were computed over 200 compounded ultrasonic images acquired over 0.4 s so that several cardiac cycles are sampled and averaged. This leads to a more efficient rejection of tissue echoes (such as arterial pulsatility). A higher frame rate could theoretically be achieved by reducing the number of images down to 50, yielding a TR of ~1 s. However, one should expect a significant SNR penalty on fUS signals due to systemic physiological noise contamination, the extent of which would require further investigations.

In conclusion, our results show that the SMS approach using a multi-array fUS probe is a very promising method for studying 3D connectomics in awake or anesthetized mice, non-invasively through the skull. Along with its seamless compatibility with awake behaving animals, we believe that this approach will pave the way for more advanced studies to help shed new light on the spatiotemporal organization of spontaneous or evoked activity of the mouse brain and its breakdown in neuropsychiatric diseases.

## Supplementary Material

Supplementary Material

## Data Availability

Data and code that support the findings of this study are available from the corresponding author upon reasonable request. Researchers wishing to obtain the raw data must contact the Office of Research Contracts at INSERM to initiate a discussion on the proposed data transfer or use.
