# Supplementary material for: High sensitivity mapping of brain-wide functional networks in awake mice using simultaneous multi-slice fUS imaging
Source: Imaging Neurosci (Camb). 2023 Nov 15;1:imag-1-00030. doi: 10.1162/imag_a_00030 (PMC12007538; doi:10.1162/imag_a_00030)
Supplement: Supplementary Material [file imag_a_00030-supp.zip › SupFig1.pdf]

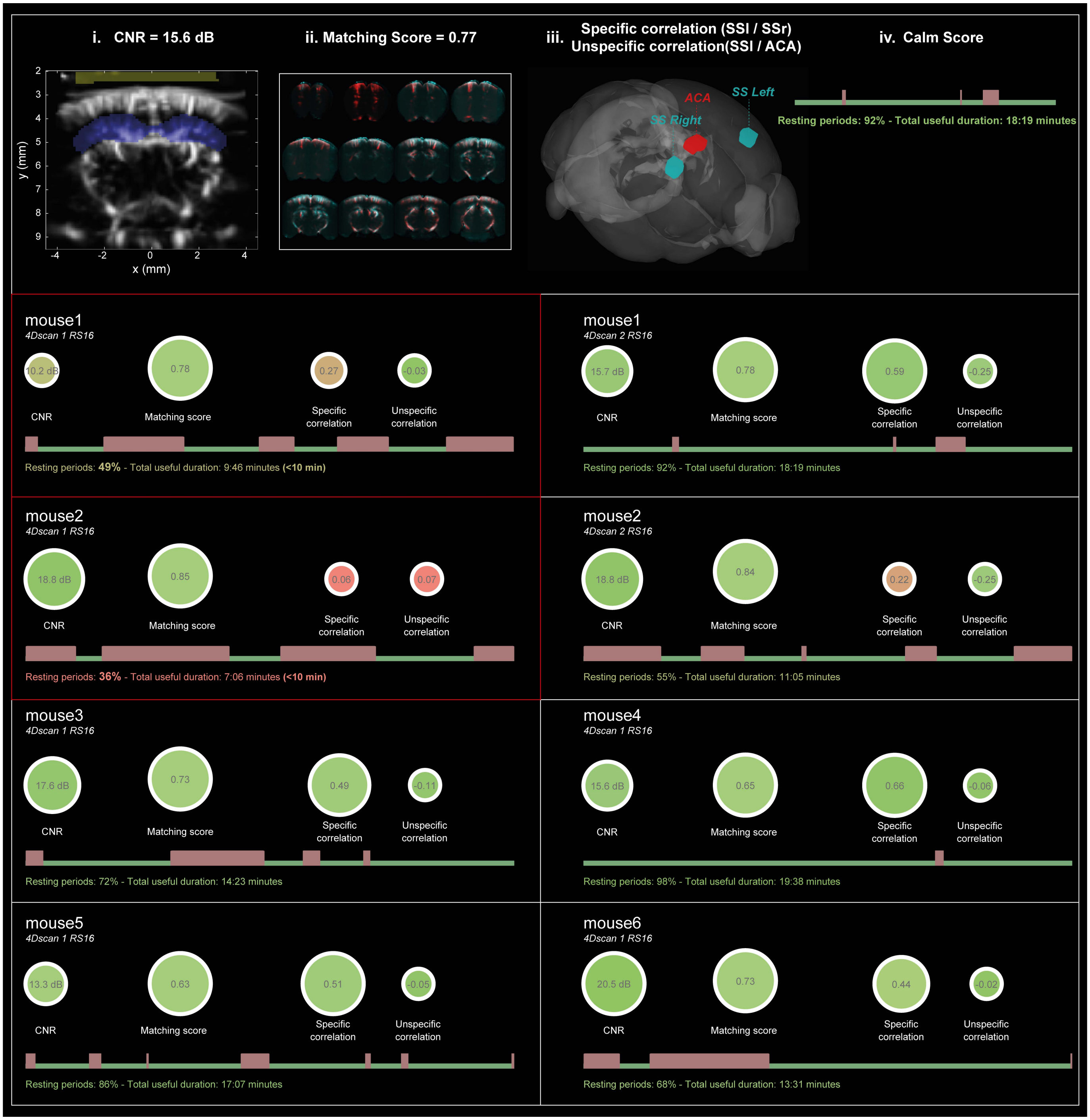

Supplementary Figure 1: Quality control metrics for each acquisition from the awake dataset. (i) The CNR is derived from the average PD signal in a ROI segmenting the hippocampal formation in one coronal slice, and a background ROI taken in the above the brain, in the acoustic gel. **(ii)** The matching score is defined as the average spatial correlation between the PD volume (green) and the average of PD across the whole awake dataset (pink). **(iii)** The correlation between two symmetric cortical regions from the somato-sensory (SS) regions characterizes specific FC, whereas the correlation between the SS cortex and the Anterior Cingulate Area (ACA) characterizes unspecific FC. **(iv)** Finally, the calm score

corresponds to the total useful duration (corresponding to resting periods) in minutes. Scans with less than 10 minutes of total useful duration were not analyzed (red rectangles).
