# Supplementary material for: High sensitivity mapping of brain-wide functional networks in awake mice using simultaneous multi-slice fUS imaging
Source: Imaging Neurosci (Camb). 2023 Nov 15;1:imag-1-00030. doi: 10.1162/imag_a_00030 (PMC12007538; doi:10.1162/imag_a_00030)
Supplement: Supplementary Material [file imag_a_00030-supp.zip › SupFig2.pdf]

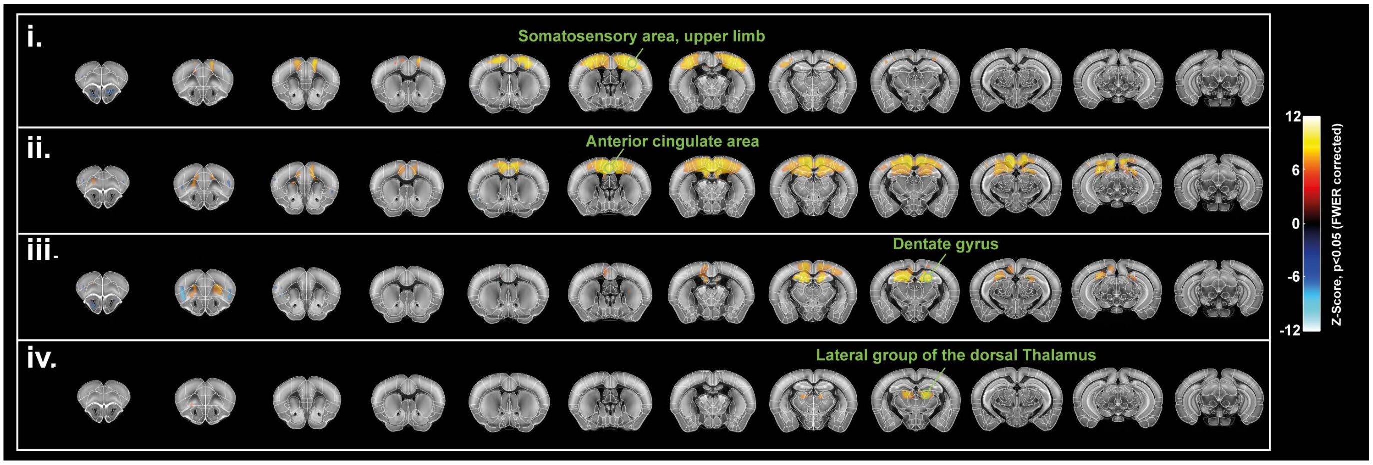

Supplementary Figure 2: Seed-based analysis in one representative mouse (n=1) reveals both cortical and subcortical functional networks. Each row represents a seed-based map, thresholded with significant connectivity (p<0.05, FWER corrected with Bonferroni procedure). Seed-based maps are represented on coronal slices overlaid with the two-photon Allen mouse template. Seed regions are denoted by the green legends.
