# Supplementary material for: High sensitivity mapping of brain-wide functional networks in awake mice using simultaneous multi-slice fUS imaging
Source: Imaging Neurosci (Camb). 2023 Nov 15;1:imag-1-00030. doi: 10.1162/imag_a_00030 (PMC12007538; doi:10.1162/imag_a_00030)
Supplement: Supplementary Material [file imag_a_00030-supp.zip › SupFig3.pdf]

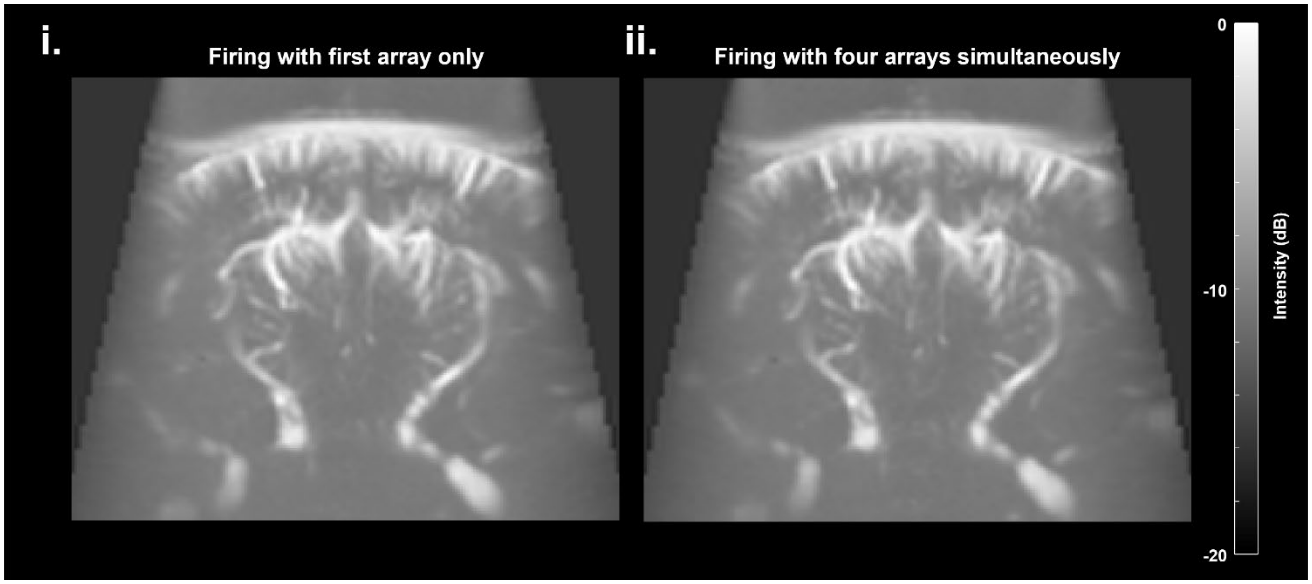

Supplementary Figure 3: Cross-talk assessment. Comparison of the images performed on the same slice on anesthetized mouse, firing with the first array of the multi-array probe only (left) and firing simultaneously with the four arrays of the multi-array probe (right). In average, the relative difference between these two images reached 0.19%, confirming that no cross-talk is occurring between the different arrays.
