# Supplementary material for: High sensitivity mapping of brain-wide functional networks in awake mice using simultaneous multi-slice fUS imaging
Source: Imaging Neurosci (Camb). 2023 Nov 15;1:imag-1-00030. doi: 10.1162/imag_a_00030 (PMC12007538; doi:10.1162/imag_a_00030)
Supplement: Supplementary Material [file imag_a_00030-supp.zip › SupTable1.pdf]

		RCA	MUX-FPM
Probe	Central Frequency (MHz)	*15*	*15*
	Number of Elements	*160 (80 + 80)*	*1024 (32 x 32)*
	Pitch (µm)	*110*	*300*
Plane Waves	N Angles	*40*	*9*
	Angular step (°)	*0.5*	*2*
	Angular sequence	*(-5°,0°); (-4.5°, 0°); ...; (4.5°,0°); (5°, 0°); (0°, -5°); (0°, -4.5°); ...; (0°, 4.5°); (0°, 5°)*	*(-2°, -2°); (-2, 0°); ((-2, 2°); (0°, -2°); (0°, 0°); (0°, 2°); (2°, -2°); (2°, 0°); (2°, 2°)*
	Voltage (V)	*25*	*25*
Temporal specs	PRF (kHz)	*20*	*12*
	MUX factor	*N/A*	*4*
	Volumerate (Hz)	*500*	*333*
	Doppler integration (s)	*2.4*	*2.4*

Supplementary table 1: RCA and MUX-FPM probe parameters, and their corresponding imaging sequence. For the MUX-FPM, the PRF was reduced to 12 kHz to prevent hardware lags due to the switching delays. As four transmissions/receptions are needed to form one volume on the whole aperture, the framerate is reduced by a factor of four.
