# Supplementary material for: High sensitivity mapping of brain-wide functional networks in awake mice using simultaneous multi-slice fUS imaging
Source: Imaging Neurosci (Camb). 2023 Nov 15;1:imag-1-00030. doi: 10.1162/imag_a_00030 (PMC12007538; doi:10.1162/imag_a_00030)
Supplement: Supplementary Material [file imag_a_00030-supp.zip › SupTable2.pdf]

Supplementary table 2: Allen Regions of Interest (ROIs) and their corresponding percentage of significantly activated voxels following visual stimulation

Color	tag	label	Side	Index	Activated voxels (%)	Number of voxels
	Brain	Brain	none	none	none	none
	Isocortex	Frontal pole, cerebral cortex	R	1	0	82
	Isocortex	Primary motor area	R	2	4,2	906
	Isocortex	Secondary motor area	R	3	18,3	1060
	Isocortex	Primary somatosensory area, nose	R	4	6,4	234
	Isocortex	Primary somatosensory area, barrel field	R	5	33,3	498
	Isocortex	Primary somatosensory area, lower limb	R	6	16,2	192
	Isocortex	Primary somatosensory area, mouth	R	7	0	507
	Isocortex	Primary somatosensory area, upper limb	R	8	4	304
	Isocortex	Primary somatosensory area, trunk	R	9	40,6	128
	Isocortex	Primary somatosensory area, unassigned	R	10	3,9	103
	Isocortex	Supplemental somatosensory area	R	11	8,1	745
	Isocortex	Gustatory areas	R	12	0	144
	Isocortex	Visceral area	R	13	0	219
	Isocortex	Auditory areas	R	14	30	454
	Isocortex	Anterolateral visual area	R	15	100	78
	Isocortex	Anteromedial visual area	R	16	92,8	69
	Isocortex	Lateral visual area	R	17	98,8	86
	Isocortex	Primary visual area	R	18	95,4	582
	Isocortex	Posterolateral visual area	R	19	96,6	59
	Isocortex	posteromedial visual area	R	20	94,1	84
	Isocortex	Laterointermediate area	R	21	94,7	38
	Isocortex	Postrhinal area	R	22	64,8	108
	Isocortex	Anterior cingulate area	R	23	76,3	414
	Isocortex	Prelimbic area	R	24	29,1	196
	Isocortex	Infralimbic area	R	25	29	69
	Isocortex	Orbital area	R	26	5,5	487
	Isocortex	Agranular insular area	R	27	0	645
	Isocortex	Retrosplenial area	R	28	91,2	849
	Isocortex	Posterior parietal association areas	R	29	86,7	181
	Isocortex	Temporal association areas	R	30	18,9	233
	Isocortex	Perirhinal area	R	31	8,8	57
	Isocortex	Ectorhinal area	R	32	5,7	122
	Isocortex	Ectorhinal area	L	33	10,3	146
	Isocortex	Perirhinal area	L	34	0	67
	Isocortex	Temporal association areas	L	35	21	248

	Isocortex	Posterior parietal association areas	L	36	76	179
	Isocortex	Retrosplenial area	L	37	91,1	854
	Isocortex	Agranular insular area	L	38	0	625
	Isocortex	Orbital area	L	39	1,6	487
	Isocortex	Infralimbic area	L	40	9,5	74
	Isocortex	Prelimbic area	L	41	16,7	204
	Isocortex	Anterior cingulate area	L	42	71,5	411
	Isocortex	Postrhinal area	L	43	41,7	96
	Isocortex	Laterointermediate area	L	44	67,7	34
	Isocortex	posteromedial visual area	L	45	83,1	83
	Isocortex	Posterolateral visual area	L	46	72,9	70
	Isocortex	Primary visual area	L	47	86,2	593
	Isocortex	Lateral visual area	L	48	95,7	92
	Isocortex	Anteromedial visual area	L	49	83,1	65
	Isocortex	Anterolateral visual area	L	50	91,3	69
	Isocortex	Auditory areas	L	51	22,4	469
	Isocortex	Visceral area	L	52	0	179
	Isocortex	Gustatory areas	L	53	0	148
	Isocortex	Supplemental somatosensory area	L	54	2,9	726
	Isocortex	Primary somatosensory area, unassigned	L	55	4	99
	Isocortex	Primary somatosensory area, trunk	L	56	14,8	128
	Isocortex	Primary somatosensory area, upper limb	L	57	2,7	299
	Isocortex	Primary somatosensory area, mouth	L	58	0	498
	Isocortex	Primary somatosensory area, lower limb	L	59	28,9	194
	Isocortex	Primary somatosensory area, barrel field	L	60	24,8	497
	Isocortex	Primary somatosensory area, nose	L	61	0	248
	Isocortex	Secondary motor area	L	62	6,3	1059
	Isocortex	Primary motor area	L	63	1,8	898
	Isocortex	Frontal pole, cerebral cortex	L	64	0	76
	Olfactory Areas	Main olfactory bulb	R	65	0,5	199
	Olfactory Areas	Anterior olfactory nucleus	R	66	0	368
	Olfactory Areas	Dorsal peduncular area	R	67	13,2	38
	Olfactory Areas	Piriform area	R	68	1	951
	Olfactory Areas	Piriform-amygdalar area	R	69	0	87
	Olfactory Areas	Postpiriform transition area	R	70	7,9	114
	Olfactory Areas	Accessory olfactory bulb	R	71	0	37
	Olfactory Areas	Taenia tecta	R	72	5,9	119
	Olfactory Areas	Nucleus of the lateral olfactory tract	R	73	0	31
	Olfactory Areas	Cortical amygdalar area	R	74	0,4	258

	Olfactory Areas	Cortical amygdalar area	L	75	7,7	259
	Olfactory Areas	Nucleus of the lateral olfactory tract	L	76	0	32
	Olfactory Areas	Taenia tecta	L	77	11,6	121
	Olfactory Areas	Accessory olfactory bulb	L	78	0	31
	Olfactory Areas	Postpiriform transition area	L	79	0	114
	Olfactory Areas	Piriform-amygdalar area	L	80	0	97
	Olfactory Areas	Piriform area	L	81	1	956
	Olfactory Areas	Dorsal peduncular area	L	82	2,5	40
	Olfactory Areas	Anterior olfactory nucleus	L	83	0	368
	Olfactory Areas	Main olfactory bulb	L	84	0	199
	Hippocampal formation	Field CA1	R	85	50,3	806
	Hippocampal formation	Field CA2	R	86	24,4	41
	Hippocampal formation	Field CA3	R	87	43,8	498
	Hippocampal formation	Fasciola cinerea	R	88	42,9	7
	Hippocampal formation	Induseum griseum	R	89	33,3	6
	Hippocampal formation	Parasubiculum	R	90	97,8	90
	Hippocampal formation	Postsubiculum	R	91	100	95
	Hippocampal formation	Presubiculum	R	92	100	32
	Hippocampal formation	Subiculum	R	93	86,5	96
	Hippocampal formation	Prosubiculum	R	94	70,8	161
	Hippocampal formation	Hippocampo-amygdalar transition area	R	95	31,7	41
	Hippocampal formation	Area prostriata	R	96	100	13
	Hippocampal formation	Dentate gyrus	R	97	66,2	571
	Hippocampal formation	Entorhinal area	R	98	34,1	932
	Hippocampal formation	Entorhinal area	L	99	19,7	941
	Hippocampal formation	Dentate gyrus	L	100	58	560
	Hippocampal formation	Area prostriata	L	101	100	10
	Hippocampal formation	Hippocampo-amygdalar transition area	L	102	37,8	37
	Hippocampal formation	Prosubiculum	L	103	37,3	158
	Hippocampal formation	Subiculum	L	104	56,9	144
	Hippocampal formation	Presubiculum	L	105	79,4	34
	Hippocampal formation	Postsubiculum	L	106	84,7	98

	Hippocampal formation	Parasubiculum	L	107	76,9	78
	Hippocampal formation	Induseum griseum	L	108	14,3	7
	Hippocampal formation	Fasciola cinerea	L	109	33,3	6
	Hippocampal formation	Field CA3	L	110	40,3	489
	Hippocampal formation	Field CA2	L	111	27,9	43
	Hippocampal formation	Field CA1	L	112	30,1	781
	CortSubplate	Claustrum	R	113	0	45
	CortSubplate	Lateral amygdalar nucleus	R	114	0	71
	CortSubplate	Posterior amygdalar nucleus	R	115	1,2	83
	CortSubplate	Endopiriform nucleus	R	116	2,7	226
	CortSubplate	Basolateral amygdalar nucleus	R	117	17,3	156
	CortSubplate	Basomedial amygdalar nucleus	R	118	1,7	117
	CortSubplate	Basomedial amygdalar nucleus	L	119	0,8	120
	CortSubplate	Basolateral amygdalar nucleus	L	120	2,7	148
	CortSubplate	Endopiriform nucleus	L	121	1,2	242
	CortSubplate	Posterior amygdalar nucleus	L	122	21,2	85
	CortSubplate	Lateral amygdalar nucleus	L	123	11,3	80
	CortSubplate	Claustrum	L	124	0	48
	Striatum	Striatum dorsal region	R	125	9,5	2060
	Striatum	Striatum ventral region	R	126	6,6	709
	Striatum	Lateral septal complex	R	127	19,3	285
	Striatum	Striatum-like amygdalar nuclei	R	128	7,6	314
	Striatum	Striatum-like amygdalar nuclei	L	129	6,5	310
	Striatum	Lateral septal complex	L	130	17,5	275
	Striatum	Striatum ventral region	L	131	2	691
	Striatum	Striatum dorsal region	L	132	6,6	2082
	Pallidium	Pallidum, dorsal region	R	133	24,7	154
	Pallidium	Pallidum, ventral region	R	134	10,9	266
	Pallidium	Pallidum, medial region	R	135	20,7	111
	Pallidium	Pallidum, caudal region	R	136	15	107
	Pallidium	Pallidum, caudal region	L	137	6,4	109
	Pallidium	Pallidum, medial region	L	138	34,1	126
	Pallidium	Pallidum, ventral region	L	139	7,3	261
	Pallidium	Pallidum, dorsal region	L	140	17,2	151
	Thalamus	Subparafascicular area	R	141	52,2	23
	Thalamus	Peripeduncular nucleus	R	142	60	5
	Thalamus	Reticular nucleus of the thalamus	R	143	81,4	113
	Thalamus	Ventral group of the dorsal thalamus	R	144	64,5	400
	Thalamus	Subparafascicular nucleus	R	145	63,2	19
	Thalamus	Geniculate group, dorsal thalamus	R	146	83	112

	Thalamus	Lateral group of the dorsal thalamus	R	147	85	234
	Thalamus	Anterior group of the dorsal thalamus	R	148	97,7	173
	Thalamus	Medial group of the dorsal thalamus	R	149	49	149
	Thalamus	Midline group of the dorsal thalamus	R	150	56,3	96
	Thalamus	Intralaminar nuclei of the dorsal thalamus	R	151	65,8	149
	Thalamus	Geniculate group, ventral thalamus	R	152	87,2	47
	Thalamus	Epithalamus	R	153	72,7	55
	Thalamus	Epithalamus	L	154	55,8	52
	Thalamus	Geniculate group, ventral thalamus	L	155	89,4	47
	Thalamus	Intralaminar nuclei of the dorsal thalamus	L	156	66,5	161
	Thalamus	Midline group of the dorsal thalamus	L	157	52,4	84
	Thalamus	Medial group of the dorsal thalamus	L	158	43,3	150
	Thalamus	Anterior group of the dorsal thalamus	L	159	81,4	172
	Thalamus	Lateral group of the dorsal thalamus	L	160	84,5	226
	Thalamus	Geniculate group, dorsal thalamus	L	161	81	116
	Thalamus	Subparafascicular nucleus	L	162	94,7	19
	Thalamus	Ventral group of the dorsal thalamus	L	163	69,9	398
	Thalamus	Reticular nucleus of the thalamus	L	164	53,5	114
	Thalamus	Peripeduncular nucleus	L	165	40	5
	Thalamus	Subparafascicular area	L	166	25	28
	Hypothalamus	Median eminence	R	167	60	5
	Hypothalamus	Periventricular zone	R	168	12,5	56
	Hypothalamus	Periventricular region	R	169	16,8	173
	Hypothalamus	Hypothalamic medial zone	R	170	23,6	310
	Hypothalamus	Hypothalamic lateral zone	R	171	36,1	491
	Hypothalamus	Hypothalamic lateral zone	L	172	36,2	473
	Hypothalamus	Hypothalamic medial zone	L	173	29,1	323
	Hypothalamus	Periventricular region	L	174	19,8	177
	Hypothalamus	Periventricular zone	L	175	4,4	46
	Hypothalamus	Median eminence	L	176	25	4
	MidBrain	Substantia nigra, reticular part	R	177	56,6	106
	MidBrain	Ventral tegmental area	R	178	63,9	36
	MidBrain	Paranigral nucleus	R	179	75	4
	MidBrain	Midbrain reticular nucleus, retrorubral area	R	180	33,3	12
	MidBrain	Midbrain reticular nucleus	R	181	29,4	408
	MidBrain	Periaqueductal gray	R	182	42,3	338

	MidBrain	Cuneiform nucleus	R	183	22,6	31
	MidBrain	Red nucleus	R	184	36,2	69
	MidBrain	Oculomotor nucleus	R	185	0	3
	MidBrain	Medial accesory oculomotor nucleus	R	186	100	1
	MidBrain	Edinger-Westphal nucleus	R	187	0	2
	MidBrain	Trochlear nucleus	R	188	50	2
	MidBrain	Paratrochlear nucleus	R	189	0	2
	MidBrain	Ventral tegmental nucleus	R	190	0	1
	MidBrain	Anterior tegmental nucleus	R	191	0	6
	MidBrain	Lateral terminal nucleus of the accessory optic tract	R	192	0	0
	MidBrain	Dorsal terminal nucleus of the accessory optic tract	R	193	0	0
	MidBrain	Medial terminal nucleus of the accessory optic tract	R	194	100	1
	MidBrain	Superior colliculus, motor related	R	195	92,8	489
	MidBrain	Pretectal region	R	196	85,2	162
	MidBrain	Pretectal region	L	197	73,5	151
	MidBrain	Superior colliculus, motor related	L	198	89	480
	MidBrain	Medial terminal nucleus of the accessory optic tract	L	199	0	3
	MidBrain	Dorsal terminal nucleus of the accessory optic tract	L	200	100	1
	MidBrain	Lateral terminal nucleus of the accessory optic tract	L	201	0	0
	MidBrain	Anterior tegmental nucleus	L	202	0	6
	MidBrain	Ventral tegmental nucleus	L	203	0	1
	MidBrain	Paratrochlear nucleus	L	204	0	2
	MidBrain	Trochlear nucleus	L	205	0	3
	MidBrain	Edinger-Westphal nucleus	L	206	0	0
	MidBrain	Medial accesory oculomotor nucleus	L	207	100	1
	MidBrain	Oculomotor nucleus	L	208	0	4
	MidBrain	Red nucleus	L	209	31,9	69
	MidBrain	Cuneiform nucleus	L	210	31,3	32
	MidBrain	Periaqueductal gray	L	211	45,4	361
	MidBrain	Midbrain reticular nucleus	L	212	33,5	412
	MidBrain	Midbrain reticular nucleus, retrorubral area	L	213	0	12
	MidBrain	Paranigral nucleus	L	214	0	2
	MidBrain	Ventral tegmental area	L	215	35	40
	MidBrain	Substantia nigra, reticular part	L	216	44,6	112
	HindBrain	Pons, sensory related	R	217	15,2	256
	HindBrain	Pons, motor related	R	218	3,2	380
	HindBrain	Pons, behavioral state related	R	219	11,3	275
	HindBrain	Medulla, sensory related	R	220	0	36
	HindBrain	Medulla, motor related	R	221	0	0

	HindBrain	Medulla, behavioral state related	R	222	0	6
	HindBrain	Medulla, behavioral state related	L	223	0	0
	HindBrain	Medulla, motor related	L	224	0	0
	HindBrain	Medulla, sensory related	L	225	9,3	43
	HindBrain	Pons, behavioral state related	L	226	12,7	259
	HindBrain	Pons, motor related	L	227	2,1	382
	HindBrain	Pons, sensory related	L	228	8,3	276
