# Supplementary material for: High sensitivity mapping of brain-wide functional networks in awake mice using simultaneous multi-slice fUS imaging
Source: Imaging Neurosci (Camb). 2023 Nov 15;1:imag-1-00030. doi: 10.1162/imag_a_00030 (PMC12007538; doi:10.1162/imag_a_00030)
Supplement: Supplementary Material [file imag_a_00030-supp.zip › SupTable3.pdf]

Supplementary table 3: Elevation extent of functional networks identified with ICA

Plausible Functional Network	Elevation extent (Nslices)	Elevation extent (mm)
DMN 1	12	6.3
DMN 2	6	3.15
LCN 1	8	4.2
LCN 2	8	4.2
LCN 3	5	2.625
VIS	5	2.625
SN	3	1.575
HIPPO 1	6	3.15
HIPPO 2	7	3.675
THAL 1	4	2.1
THAL 2	3	1.575
MIDB 1	3	1.575
MIDB 2	2	1.05
BF	4	2.1
OLF	2	1.05
AMYG	5	2.625
